# Students’ experiences with remote learning during the COVID-19 school closure: implications for mathematics education

**DOI:** 10.1016/j.heliyon.2021.e07523

**Published:** 2021-07-10

**Authors:** Angel Mukuka, Overson Shumba, Henry M. Mulenga

**Affiliations:** a African Centre of Excellence for Innovative Teaching and Learning Mathematics and Science, University of Rwanda-College of Education, Rwanda; b Department of Mathematics, Science, and Technology Education, Mukuba University, Kitwe, Zambia; c School of Mathematics and Natural Sciences, Copperbelt University, Kitwe, Zambia

**Keywords:** COVID-19, Remote learning, Mathematics education, ICT

## Abstract

This paper reports the findings of a descriptive survey research that explored secondary school students' experiences with mathematics remote learning during the Corona Virus Disease 2019 (COVID-19) school closure. The study involved 367 students of ages 13 to 21 selected from six secondary schools in Kitwe district of Zambia using the cluster random sampling method. Using a mixed-methods research approach, quantitative and qualitative data were merged to provide a comprehensive analysis of the main findings in the context of the existing literature, the government's response to COVID-19 school closure, and the challenges associated with remote learning during that time. Research findings show that more than 56% of the respondents did not have sufficient access to Information and Communication Technologies (ICT), electricity, and internet services. Most of these respondents also held a belief that mathematics is a subject that is best learned with face-to-face interactions between the teacher and students, and among students. These results suggest a need for the education systems in Zambia and other similar contexts to put up infrastructure that supports the blended and online learning models during and after the COVID-19 pandemic.

## 1 Introduction

The coronavirus disease 2019 (COVID-19) has been considered one of the greatest health threats to humanity worldwide. As of March 8, 2021, the World Health Organisation (WHO) had recorded a cumulative total of 116,521,281 confirmed cases of COVID-19 with 2,589,548 as a cumulative total of deaths from over 215 countries or territories globally (World Health Organisation, 2021). It all started on December 31, 2019, when the Wuhan Municipal Health Commission in China, reported a cluster of cases of pneumonia after which a novel coronavirus was identified. As the disease continued to spread across the globe at an alarming rate it became recognized by WHO as a pandemic on March 11, 2020 (World Health Organisation, 2020). Consequently, many countries decided to close schools and universities as one of the measures to minimize person-to-person transmission. This closure posed a serious threat to educational provision worldwide.

According to the Global Education Coalition launched by United Nations Educational, Scientific, and Cultural Organization (UNESCO), over 186 countries worldwide faced nationwide or partial closures of educational institutions including schools, colleges, and universities. More than 1.6 billion learners, representing nearly 80% of the world's student population in primary and secondary schools were affected by the school closures (UNESCO, 2020). The school closures led to challenges that included interrupted learning, lack of proper nutrition among some learners, higher drop-out rates, and lowered academic achievement levels, among others (Ahedor, 2020; Lancker and Parolin, 2020; Sintema, 2020; UNESCO, 2020). The closure of schools inspired many education systems world-over to adopt remote teaching and learning.

According to Ray (2020), remote learning provides an opportunity for students and teachers to remain connected and engaged with the content while working from their homes. In the context of this study, all forms of learning that students' experienced during the COVID-19 school closure are referred to as remote learning opportunities. From the Zambian experience, these include students' self-study using both electronic and hard copy learning materials, online learning (e.g., the smart revision, and e-learning portals, social media, etc.), lessons broadcasted by radio or television, and private tuitions provided by teachers. However, it had been anticipated that remote teaching and learning had added to the challenge of learning mathematics, a subject perceived to be difficult by many learners at the secondary level of education in Zambia. As a result, it became important to explore secondary school students’ experiences with mathematics remote learning during the COVID-19 school closure.

While recognizing the fact that the entire education system is affected, special attention in this study has been given to mathematics. This is because students’ performance in mathematics in Zambia has been extremely low even before the COVID-19 outbreak (Examinations Council of Zambia, 2012; 2015; 2016; 2018; Mukuka et al., 2019). In that regard, questions about the type of teaching practices that would be useful during and after the COVID-19 pandemic are worth exploring. While there might be no straightforward answers to such questions, this paper brings out some suggestions (based on the data collected, existing literature, and personal experiences) on some potentially effective mathematics teaching and learning practices during the current situation and beyond.

## 2 Context of the study

Zambia and many other countries/regions worldwide have witnessed tremendous shocks resulting from the COVID-19 induced crisis. Since its inception in Zambia, the COVID-19 pandemic was anticipated to worsen the problem of students’ low achievement levels especially in STEM subjects (Sintema, 2020). The first two positive cases of COVID-19 in Zambia were announced on March 18, 2020, by the Minister of Health during the daily updates on the COVID-19 situation in the country. This announcement was preceded by a press statement a day earlier in which the government through the Minister of Health ordered the closure of all schools and universities by March 20, 2020, in a bid to combat the spread of the disease. The cumulative total of COVID-19 positive cases in Zambia had risen to 82,655 with 78,721 recoveries and 1,132 deaths as of March 9, 2021 (Worldometer, 2021).

At the onset of school closures, this outbreak reminded us of the need for alternative mathematics teaching practices outside the classroom environment. The COVID-19 pandemic forced an immediate switch to emergency remote learning mode. In conjunction with the Ministry of General Education (MOGE), an interactive e-learning portal and smart revision platform was launched by the Zambia Telecommunications Company (ZAMTEL) in partnership with the Examinations Council of Zambia (ECZ). The e-learning portal can be accessed at www.elearning.co.zm while smart revision is accessible at www.smartrevision.co.zm. Access to these platforms has been made free of charge during the current COVID-19 outbreak period. The national e-learning portal hosts various teaching and learning materials including e-books, links for specialized services, and a virtual library that can be useful to both teachers and students. The smart revision portal, on the other hand, hosts ECZ past examination papers alongside sample answers and revision tips for grades 7, 9, and 12. Besides that, the ministry of general education in partnership with the Zambia National Broadcasting Corporation (ZNBC) had established a television (TV) channel dedicated to broadcasting both primary and secondary school lessons in all subjects. These lessons are being broadcasted even after the re-opening of schools.

Being aware that a larger proportion of the school-going children did not have access to these platforms, MOGE had also engaged various local radio stations in broadcasting these lessons. This was also done as part of the measures for attaining one of the most ambitious Sustainable Development Goals (SDGs) – “*Inclusive and equitable quality education”*. The Ministry of General Education had also partnered with UNICEF on the printing of educational materials for learners. Some teachers especially those from urban schools had been collaborating with parents to form Whatsapp groups through which they could reach out to the students by giving them assignments, providing feedback, and attending to individual learners’ needs. It was expected that a successful implementation of the measures highlighted above was bound to yield positive results not only for the present situation but also for the future.

With this background, it was necessary to undertake an empirical investigation whose aim was to find out students’ experiences with remote learning of mathematics during the COVID-19 school closure. This study was guided by three research questions:

i. How did students access mathematics lessons during the COVID-19 school closure in selected secondary schools of Kitwe district, Zambia?
ii. What benefits were associated with the mathematical learning options that were used by secondary school students during the COVID-19 school closure?
iii. What challenges did students face with mathematics remote learning during the COVID-19 school closure?

Answering these questions provided insights into what ought to be done to improve mathematics remote learning in times of the COVID-19 outbreak and beyond.

## 3 Method

### 3.1 Research design

A descriptive survey research design was employed. This design was appropriate for the study because it enabled the researchers to collect both qualitative and quantitative data at once without close or prolonged contact with respondents in adherence to COVID-19 social distancing measures. It was further assumed that this design would enable the researchers to get detailed views from respondents regarding their experiences with mathematics remote learning during the COVID-19 school closure. Data to address the research questions were collected from September 28, 2020, to October 23, 2020. This was soon after the presidential directive to re-open schools at all levels of education, with much emphasis on strict adherence to COVID-19 preventive measures as stipulated by the World Health Organisation and the country's Ministry of Health.

### 3.2 Study participants

A cluster random sampling method was used to select the research participants from the target population involving grade 10 and grade 11 students from public secondary schools within Kitwe district. To ensure that both the urban and peri-urban students were included in the sample, cluster sampling was the most appropriate for the study. Fraenkel et al. (2006, p. 95) also justified that this type of sampling is one of the easiest to implement in schools, and it consumes less time. Based on the information obtained from the district education office, these schools were categorized into two clusters of their geographical location (urban versus peri-urban). In our classification, we considered a school to be urban if it is located within a 10km-radius from the central business district (CBD).^1^ This implies that all the schools located outside the 10km radius of the CBD were classified as peri-urban.

Thereafter, three schools were randomly selected from each cluster bringing the total number of participating schools to 6. From each selected school, two classes (one grade 10, and one grade 11) were randomly selected. This means that a total of 12 classes (6 grade 10, and 6 grade 11) participated in the study. Finally, all the students from each selected class were included in the sample bringing the total number of respondents to 367 of which 172 were from urban schools while 191 came from peri-urban schools. In terms of gender, 178 (48.5%) were male while 189 (51.5%) were female. The ages of respondents ranged from 13 to 21 (*M* = 16.92, *SD* = 1.47). It was further noted that 174 (47.4%) respondents were in their 10^th^ grade while 193 (52.6%) were doing grade 11. Grade 10 and 11 students were targeted because they were non-examination classes and they stayed at home during the lockdown longer than the examination classes (grades 9 and 12). Grade 8 students were excluded because they only had less than 3 months of secondary school experience at the time of the school closure.

### 3.3 Instrumentation

A semi-structured questionnaire, involving both closed-ended and open-ended questions was administered to the respondents. The questionnaire was the only data collection instrument used as it was deemed appropriate for avoiding researchers' close and/or prolonged contact with respondents for strict adherence to the “social distancing” COVID-19 preventive measure. The formulation of the questionnaire items was done in line with the existing literature on prospects of mathematics education during the COVID-19 pandemic (Bakker and Wagner, 2020; Engelbrecht et al., 2020a, 2020b; Olivier, 2020). For instance, the above-cited studies had raised some concerns about online lesson delivery, the associated benefits as well as the challenges that may arise due to the lack of ICT services in some settings. The formulation of questionnaire items for this study was also inspired by the government's response to COVID-19 school closure and the anticipated challenges associated with remote learning in Zambia.

Prior to the main data collection, a draft questionnaire was sent to the experts for validation. These experts comprised 2 Ph.D. students in mathematics education, 2 master's students in mathematics education, 6 college/university lecturers in mathematics and science education, and 5 secondary school teachers of mathematics. These validators were selected because of their vast experience with mathematics education research, and/or their vast experience with the Zambian secondary school mathematics curriculum. They were asked to comment on the quality of the included items in terms of sufficiency, relevance, clarity, and coherence. After getting feedback from these validators, their suggestions and comments were analyzed and the final questionnaire was developed.

The questionnaire comprised three sections namely, demographic information, students’ access to mathematics learning during the COVID-19 school closure, and the challenges associated with the available mathematics learning options during that time. After questionnaire refinement, data collection in all the sampled schools/classrooms commenced. The questionnaire and the associated datasets are openly available at https://doi.org/10.17632/mb8sdf576c.1.

### 3.4 Data analysis

Descriptive statistics such as frequency distributions were used to analyze data. This gave a provision for quantifying the responses for the closed-ended questionnaire items. Since the respondents came from two different clusters, a Chi-square test was used to determine whether certain factors affecting remote learning during that period could be associated with the schools’ geographical location. Both the frequency distributions and Chi-square tests were generated using the Statistical Package for Social Sciences (SPSS) version 21. The analysis of data from open-ended questionnaire items did not strictly adhere to all procedures of qualitative data analysis entailing coding, categorizing, and thematizing (Nowell et al., 2017). Instead, excerpts from open-ended responses were selected that were judged to be consistent and illustrative of closed-ended questionnaire responses.

### 3.5 Ethical considerations

Research ethics were upheld at all stages of the study. First of all, permission from the district education office was sought and granted. Before distributing the questionnaires to the students, the headteacher or deputy headteacher for each participating school had to provide advice on the appropriate time to engage with selected students, while adhering to COVID-19 preventive measures. Before distributing the questionnaires to the students, the purpose of the study was explained to them and they were free to participate or not. Besides that, no name of the school, name of a class, or name of a respondent has been disclosed in any publication except for the name of the district/city where the research was conducted.

## 4 Results

### 4.1 Mathematics learning options during the COVID-19 school closure

Students were asked to select the learning options that were accessible to them during the COVID-19 school closure, to indicate the most used, and to justify their preference. Table 1 displays their choices. Differences in row totals (N) are attributed to the fact that not all the 367 students responded to all the questionnaire items.

**Table 1:** Accessible mathematics learning options during the COVID-19 school closure.

How were you learning mathematics during the COVID-19 school closure?	School Type	Number of Learners (*N*)	Response	
			Yes N (%)	No N (%)
1. Self-study using hard copies of mathematics textbooks, notebooks, etc.	Urban	175	145 (82.9)	30 (17.1)
	Peri-urban	191	141 (73.8)	50 (26.2)
	Total	366	286 (78.1)	80 (21.9)
2. Self-study using e-Learning and Smart Revision portals	Urban	170	44 (25.9)	126 (74.1)
	Peri-urban	187	34 (18.2)	153 (81.8)
	Total	357	78 (21.8)	279 (78.2)
3. Televised mathematics lessons on ZNBC's TV4 channel	Urban	176	88 (50.0)	88 (50.0)
	Peri-urban	191	71 (37.2)	120 (62.8)
	Total	367	159 (43.3)	208 (56.7)
4. Mathematics lessons aired on the radio	Urban	172	10 (5.8)	162 (94.2)
	Peri-urban	185	8 (4.3)	177 (95.7)
	Total	357	18 (5.0)	339 (95.0)
5. Private lessons provided by a mathematics teacher at home	Urban	174	70 (40.2)	104 (59.8)
	Peri-urban	189	54 (28.6)	135 (71.4)
	Total	363	124 (34.2)	239 (65.8)
6. Online lessons that were provided by mathematics teachers.	Urban	174	37 (21.3)	137 (78.7)
	Peri-urban	191	35 (18.3)	156 (81.7)
	Total	365	72 (19.7)	293 (80.3)

***Note.*** The numbers indicated in brackets are the corresponding percentages based on the sample size, *N,* or the row totals.

Results displayed in Table 1 reflect that self-study using hard copies of various learning resources was the most used (78.1%) mathematics learning option among all the respondents. This was followed by the televised mathematics lessons that were used by 43.3% of the respondents, while private lessons provided by mathematics teachers at home (34.2%) were the third most used learning mode.

Results further revealed that 21.8% of the respondents (25.9% urban and 18.2% peri-urban students) used the recently launched e-learning and smart revision portals, while only 19.7% were able to learn mathematics through lessons provided by their teachers online. The least used learning option for both urban (5.8%) and peri-urban (4.3%) was through the lessons broadcasted by the radio. Based on findings displayed in Table 1, it appears that more urban students had access to digitalized learning options such as lessons broadcasted on TV, e-learning and smart revision portals, and online learning through Whatsapp, and Facebook, than their counterparts in the peri-urban areas.

Self-study using hard copies of various learning resources was not only the most accessible but was also rated as the most used mathematics learning option during the COVID-19 school closure. Results showed that 48.3% of the urban students and 46.6% of the peri-urban students rated this mode of learning as the most used during the said period. When students were asked to justify their preferences, it was revealed that this learning option was not only the cheapest way to learn mathematics but was also the most convenient one since students were able to study at any time they felt a need to do so. Besides that, access to this learning option was not affected by the geographical location of the respondent's school, neither was it affected by most of the factors itemized in Table 2. Below we quote some of the actual reasons within this category of respondents:

***Respondent 39****: Since my parents cannot afford to buy a TV set or pay for extra lessons, studying on my own was a better and easier way to learn mathematics*

***Respondent 44:****Because the more you do things on your own the more you know*

***Respondent 305****: Because I did not have a chance to learn mathematics using any other way apart from studying on my own using textbooks and my class notes.*

**Table 2:** Factors affecting students mathematical learning during the COVID-19 school closure.

Factors	School Type	Number of Learners (*N*)	Response	
			Affected	Not Affected
1. Lack of electricity	Urban	176	100 (56.8)	76 (43.2)
	Peri-urban	191	130 (68.1)	61 (31.9)
	Total	367	230 (62.7)	137 (37.3)
2. Irregular supply of electricity	Urban	175	131 (74.9)	44 (25.1)
	Peri-urban	190	161 (84.7)	29 (15.3)
	Total	365	292 (80.0)	73 (20.0)
3. Lack of television (TV) set	Urban	173	54 (31.2)	119 (68.8)
	Peri-urban	191	94 (49.2)	97 (50.8)
	Total	364	148 (40.7)	216 (59.3)
4. Lack of a radio	Urban	173	54 (31.2)	119 (68.8)
	Peri-urban	184	79 (42.9)	105 (57.1)
	Total	357	133 (37.3)	224 (62.7)
5. Lack of ICT gadgets like smartphones, computers, etc.	Urban	169	114 (67.5)	55 (32.5)
	Peri-urban	190	131 (68.9)	59 (31.1)
	Total	359	245 (68.2)	114 (31.8)
6. Irregular TV channel subscriptions	Urban	173	94 (54.3)	79 (45.7)
	Peri-urban	185	114 (61.6)	71 (38.4)
	Total	358	208 (58.1)	150 (41.9)
7. Lack of mathematics textbooks and other learning materials	Urban	173	140 (80.9)	33 (19.1)
	Peri-urban	190	146 (76.8)	44 (23.2)
	Total	363	286 (78.8)	77 (21.2)
8. Lack of a more knowledgeable person to explain certain mathematical concepts	Urban	175	137 (78.3)	38 (21.7)
	Peri-urban	191	150 (78.5)	41 (25.5)
	Total	366	287 (78.4)	79 (21.6)
9. Lack of internet access	Urban	174	110 (63.2)	64 (38.8)
	Peri-urban	189	141 (74.6)	48 (25.4)
	Total	363	251 (69.1)	112 (30.9)
10. Limited access to the internet	Urban	173	124 (71.7)	49 (28.3)
	Peri-urban	191	154 (80.6)	37 (19.4)
	Total	364	278 (76.4)	86 (23.6)

***Note.*** The numbers indicated in brackets are the corresponding percentages based on the total number of responses for each factor (row total or sample size).

On the other hand, some students did not use this mode of learning indicating that they had challenges whenever they failed to understand certain concepts since there was no one to ask for clarifications. They indicated that this learning option was only used when revising previously understood mathematical concepts. One of the respondents gave the following reason:

***Respondent 63****: Studying mathematics on my own was not good. I was separated from my friends and my teacher because of social distancing and I had no one to ask about things I did not understand*

Most respondents who held this belief emphasized that mathematics is a subject that is best learned with face-to-face interactions between the teacher and students, and among students.

Concerning mathematics lessons broadcasted on TV, 19.3% of the urban students and 13.1% of the peri-urban students indicated that it was the most used learning mode during the COVID-19 school closure. This group of respondents stressed that this learning mode needed to continue even after the COVID-19 imposed lockdown because it helped them to fully understand mathematical concepts that they did not understand during their physical class sessions with their teachers. Nevertheless, this learning option was less popular among the peri-urban students compared to their counterparts from urban schools. Another shortcoming associated with the lessons broadcasted on TV was that students did not have a chance to ask for clarifications. This view is reflected in the following submission by one of the respondents:

***Respondent 114****: Although it was good to learn on TV during the COVID-19 lockdown, I would like to learn at school, where I can ask for clarifications when I fail to understand something.*

Private lessons provided by mathematics teachers to students in their respective homes was another learning option that was preferred by a substantial number of students from both urban schools (15.4%) and peri-urban schools (9.4%). Unlike the ‘self-study, and the televised lessons’ learning options, the majority of students who preferred private lessons indicated that a teacher needed to be around to provide clarifications whenever students faced some difficulties in understanding certain concepts. The following quotes of students provided the reasons regarding their preference of this learning option:

***Respondent 293****: I understand mathematics more when there is someone to explain and correct me where I go wrong.*

***Respondent 358****: Private lessons provided by mathematics teachers at home were beneficial to me because I was able to ask where I was wrong and where I did not understand.*

These responses suggest that some students still believe that learning mathematics can only be possible when there is someone around to guide and correct them. Over-dependence on teachers as the sole providers of mathematical knowledge could also be attributed to students’ lack of self-confidence.

The least used mode of learning among both the urban (1.1%) and the peri-urban (0.5%) students was learning by listening to the lessons broadcasted by the radio. This confirms our earlier findings in Table 1 that only 5% of all the respondents reported having used this mode of learning. Finally, 2.2% of the urban students and 20.9% of the peri-urban students reported that none of the learning options itemized in Table 1 was useful to them. The number appears to be quite high among the peri-urban students due to the lack or limited access to some of the learning options that were available to their counterparts from the urban communities. The majority of these respondents indicated that they found none of those learning methods useful because mathematics was generally difficult for them. The following were written by these students to justify why none of those learning options was useful to them:

***Respondent 262***: *None of the above because I don't even like maths. So I was not doing anything in mathematics.*

***Respondent 339***: *I enjoyed none because I can't understand many things on my own. I need some teachers to help me and I can't even concentrate when I am studying mathematics alone.*

The above narrations appear to indicate that students’ negative attitude towards mathematics and dependency on the teacher as the major source of knowledge might have hampered their mathematics remote learning experiences.

### 4.2 Challenges associated with remote learning during the COVID-19 school closure

Students’ choices of the most useful learning options could have been influenced by many factors. To that effect, students were asked to indicate whether, or not each of the itemized factors (Table 2) affected their mathematical learning during the COVID-19 school closure. Since not all students responded to all the questionnaire items, the role totals in Table 2 are not the same.

It has been observed, from Table 2 results that the number of students who were affected by 8 of the 10 factors was more than the number of students who indicated that they were not affected. Only the lack of a television set and lack of radio seemed to have affected a smaller number of students compared to those who were not affected. In terms of the geographical location of the sampled schools, results in Table 2 indicate that more students from peri-urban schools were affected by each of the 10 factors compared to their counterparts from urban schools. This could be attributed to the fact that urban students had more access to various learning options than their counterparts from peri-urban schools.

To establish whether this association was significant or not, a Pearson Chi-square test was conducted for each of the factors listed in Table 2. The Pearson Chi-square results are displayed in Table 3.

**Table 3:** Pearson Chi-square tests of Association between School Type and each of the Factors.

Factors	Chi-square Value (*χ*^2^)	df	Asymp. Sig. (2-sided)
1. Lack of electricity	4.951^a^	1	.026
2. Irregular supply of electricity	5.557^a^	1	.018
3. Lack of television (TV) set	12.191^a^	1	.000
4. Lack of a radio	5.240^a^	1	.022
5. Lack of ICT gadgets like smartphones, computers, etc.	.092^a^	1	.762
6. Irregular TV channel subscriptions	1.950^a^	1	.163
7. Lack of mathematics textbooks and other learning materials	.903^a^	1	.342
8. Lack of someone to explain certain mathematical concepts	.003^a^	1	.954
9. Lack of internet access	5.504^a^	1	.019
10. Limited access to the internet	4.032^a^	1	.045

a 0 cells (0.0%) have an expected count less than 5. The minimum expected count is 36.70.

Based on the results displayed in Table 3, there were no statistically significant associations between each of the factors (5), (6), (7), and (8), and the schools’ geographical location. This may imply that while more peri-urban students were affected by lack of ICT gadgets, irregular TV channel subscriptions, lack of mathematics textbooks, and lack of someone to explain certain mathematical concepts, results show that this association was not significant. This may also imply that the way these factors affected the peri-urban schools was not significantly different from the way they affected the urban students. This suggests that while a substantial number of students from all the sampled schools were affected by these factors, not many differences were spotted based on the geographical location of schools to which these students belonged.

On the other hand, there were statistically significant associations between students’ school geographical location, and each of the factors such as lack of electricity [*χ*^*2*^ (1) = 4.951, *p* = .026], irregular supply of electricity [*χ*^*2*^ (1) = 5.557, *p* = .018], lack of TV [*χ*^*2*^ (1) = 12.191, *p* < .0001], lack of a radio [*χ*^*2*^ (1) = 5.240, *p* = .022], lack of access to the internet [*χ*^*2*^ (1) = 5.504, *p* = .019], and limited access to the internet [*χ*^*2*^ (1) = 4.032, *p* < .045]. This may imply that each of these factors had affected the peri-urban students more than the urban students.

## 5 Discussion

This study explored secondary school students’ experiences with mathematics remote learning during the COVID-19 school closure. A switch to a remote learning option during the COVID-19 school closure was good for the continuous provision of education to all learners of school mathematics and other subjects. However, the findings of this study have provided evidence that the implementation of such a measure might have been hampered by some challenges. Besides, the reported challenges do not seem to be unique to the Zambian context. For instance, a study conducted in Jordan by Abuhammad (2020) also cited personal, logistical, and technical barriers regarding the distance learning mode during the COVID-19 lockdown. Another study conducted in Bangladesh by Al-Amin et al. (2021) reported that limited access to the internet and electricity were among the major impediments to remote learning in most developing countries.

While educators from some technologically advanced countries might be managing to reach out to their learners through an online mode of lesson delivery, some low-income countries may find remote learning quite difficult (Ahedor, 2020; Camacho-Zuñiga et al., 2021; Oyediran et al., 2020). This could be attributed to limited resources by most schools and a lack of experience by the vast majority of teachers with online teaching modes. Similarly, Olivier (2020) indicated that an online mode of lesson delivery may not favour schools with limited resources whose teachers may not be sufficiently skilled and motivated. While acknowledging that students with access to ICT gadgets and internet services may not be the majority we argue that COVID-19 school closure in Zambia, and elsewhere could be a wake-up call for education systems to put up infrastructure that supports the blended and online learning modes. The provision of ICT products and services is bound to make the teaching of mathematics easier, both remotely and during physical classroom interactions.

In line with the foregoing, the big question that requires an answer is how we can ensure that our students continue learning mathematics amidst the COVID-19 school closures and other future calamities. Based on the challenges associated with the effective and successful implementation of the measures discussed in this paper, it seems almost impossible to reach out to all learners of school mathematics especially those in rural areas and other underprivileged communities. Nevertheless, suggestions on the possible solutions to some of the highlighted challenges are given:

First, education providers should always consider the social and economic status of learners when designing instructional strategies. Here we concur with Sehoole (2020) who has challenged the education systems worldwide to take advantage of the COVID-19 outbreak to bridge the gap between the rich and the poor in terms of access to quality education. It is also worth noting that most of the challenges highlighted here are not unique to the Zambian context. For instance, Zhang et al. (2020) also report that “the weakness of the online teaching infrastructure, the inexperience of teachers (including unequal learning outcomes caused by teachers’ varied experience), the information gap, and the complex home environment” hurt student learning during the COVID-19 lockdown.

Second is a need to provide ICT products and services to all learners regardless of their socio-cultural and economic status. If such products and services are not provided to all then our students in the rural and other underprivileged communities will lag. This means that the gap between those who might have access to these services and those who do not have access will widen, yet all students will be subjected to the same examinations and later on compete for the same jobs on the labour market. In their systematic review of 22 studies, Verschaffel et al. (2019), also found that multimedia and computer-assisted collaborative learning environments showed positive effects on mathematical and metacognitive learning outcomes. Nevertheless, we are aware that providing access to ICT services to all students is not attainable within a short period because it requires a lot of resources from the government and other stakeholders. Because of this, schools that are unable to provide e-learning services to their students may scale up the printing of study materials and devise a mechanism for the distribution of such materials to their students. Despite being a good alternative as it is sustainable, printing, and distribution of these materials may also come at a great cost. The other challenge that comes with this undertaking is ensuring that the printed materials are easy for students to understand. There may be no immediate solutions to this but there is a need to consider training teachers at different levels of mathematics education on how to prepare mathematics teaching and learning materials that can easily be understood by all the learners.

The third point is a need for each school to design its virtual and/or a physical mathematics laboratory that is fully equipped with learning materials, mathematical games, and various teaching and learning aids. Social media platforms such as Facebook, Whatsapp, Twitter, and so forth where teachers and students can interact could be established in all schools. Mathematical games and higher-order thinking questions that require students to conjecture, justify, and mathematize (Mukuka et al., 2020a; 2020b; Ukobizaba et al., 2021) could be posted on such platforms to enhance students’ understanding of various mathematical concepts, and problem-solving skills.

Fourth, effective implementation of the highlighted interventions requires a reasonable level of expertise by teachers. In this vein, we concur with Zhang et al. (2020) that “equipping teachers with relevant skills on e-learning platforms, through professional development, with legal, financial, and administrative support from the government, becomes crucial” (p.5). Similarly, Barakabitze et al. (2019) have stressed the need for African countries to engage in intensive ICT skills training for teachers to achieve the United Nations Sustainable Development Goal (SDG) of “ensuring inclusive and quality education for all and promote lifelong learning” on top of improving the quality of teaching and learning STEM subjects. This could be attributed to the fact that teacher effects on students’ mathematical achievement account for approximately 34% (Kyriakides and Creemers, 2009). Additionally, Kyriakides et al. (2013) argued that “without effective teacher guidance and instruction in the classroom, learning cannot be achieved” (p.143). This suggests that a teacher is one of the significant determinants of student learning. This is why it is important to orient teachers on lesson planning and delivery as schools transition to remote learning during the COVID-19 pandemic and beyond.

Last, students' negative attitudes, low self-confidence, and low motivation towards mathematics have been stated by some of the students in this research. It has also been established that most students hold a belief that they cannot learn mathematics effectively without teacher guidance in a face-to-face environment. This demonstrates a need for interventions that are aimed at boosting students’ confidence and motivation to learn mathematics even beyond the physical classroom environment. Mathematics has always been offered as a compulsory subject for all learners from kindergarten to upper secondary levels of the Zambian education system. This is because, by the time these students leave school, they should be able to demonstrate clear mathematical thinking and mathematical knowledge in solving real-world problems (Curriculum Development Centre, 2013). This leaves teachers and other stakeholders in mathematics education with no other option than to ensure that students develop a positive attitude towards mathematics as it is one of the key attributes for meaningful mathematical learning.

### 5.1 Study limitations

One major limitation of this study is that the questionnaire was the only research instrument used. While an explanation to this methodological limitation has been given, it suffices to point out that not all the required information might have been gathered as some responses needed some follow-up questions through interviews or other forms of data collection. Another limitation of the study was that only one district was involved. While the contexts of other districts in Zambia may not differ significantly from that of Kitwe, this methodological limitation makes it difficult to generalize the research findings to other contexts especially those that are completely rural.

Given these limitations, it is recommended that future studies on this subject should increase the number of research participants by increasing the number of districts and secondary schools. Mixed methods studies may provide further insights that might have not been captured in the present study. There is also a need for future studies to provide further insights on how ICT can promote the teaching of mathematics during and after the COVID-19 pandemic.

## 6 Conclusion

One of the key findings of this study is that although peri-urban students experienced more difficulties in accessing remote learning during the COVID-19 school closure, urban students equally experienced some challenges including lack of access to ICT services, irregular supply of electricity, and lack of motivation to learn without physical interaction with the teacher and fellow students and their lack of self-confidence. There is, therefore, a need for secondary schools, with the help of the government and other stakeholders to promote the establishment of e-learning facilities countrywide. It is also important to note that the suggestions given in this paper are just the tip of the iceberg. There is a need for concerted efforts among the teachers, the Zambia Association for Mathematics Education (ZAME), MOGE, ECZ, parents, and other stakeholders to ensure that all school-going children are provided with quality mathematics education during and after the COVID-19 pandemic. To ensure that systematic and sustainable solutions are provided, all factors that affect access to education during a crisis like the COVID-19 pandemic should be explored empirically and documented to provide a basis for further actions, in policy, theory, and practice. Since one of the important findings of this study refers to the students’ dependence on the teacher, they should be encouraged to learn mathematics on their own through various platforms such as the ones that have been highlighted in this paper.

## Declarations

### Author contribution statement

Angel Mukuka, Overson Shumba, Henry M. Mulenga: Conceived and designed the experiments; Performed the experiments; Analyzed and interpreted the data; Contributed reagents, materials, analysis tools or data; Wrote the paper.

### Funding statement

This research did not receive any specific grant from funding agencies in the public, commercial, or not-for-profit sectors.

### Data availability statement

Data associated with this study has been deposited at Mendeley Data https://doi.org/10.17632/mb8sdf576c.1.

### Declaration of interests statement

The authors declare no conflict of interest.

### Additional information

Supplementary content related to this article has been published online at Data In Brief.

## Footnotes

1 Central business district (CBD) refers to commercial and business centre of Kitwe city.
